# XDose: toward online cross-validation of experimental and computational X-ray dose estimation

**DOI:** 10.1007/s11548-020-02298-6

**Published:** 2020-12-04

**Authors:** Philipp Roser, Annette Birkhold, Alexander Preuhs, Philipp Ochs, Elizaveta Stepina, Norbert Strobel, Markus Kowarschik, Rebecca Fahrig, Andreas Maier

**Affiliations:** 1 Pattern Recognition Lab, Friedrich-Alexander Universität Erlangen-Nürnberg, 91058 Erlangen, Germany; 2 Innovation, Advanced Therapies, Siemens Healthcare GmbH, 91301 Forchheim, Germany; 3 Erlangen Graduate School in Advanced Optical Technologies (SAOT), Friedrich-Alexander Universität Erlangen-Nürnberg, 91052 Erlangen, Germany; 4 Institute of Medical Engineering Schweinfurt, University of Applied Sciences Würzburg–Schweinfurt, 97421 Schweinfurt, Germany

**Keywords:** Anthropomorphic phantom, Dosimetry, MOSFET, Monte Carlo simulation

## Abstract

**Purpose:**

As the spectrum of X-ray procedures has increased both for diagnostic and for interventional cases, more attention is paid to X-ray dose management. While the medical benefit to the patient outweighs the risk of radiation injuries in almost all cases, reproducible studies on organ dose values help to plan preventive measures helping both patient as well as staff. Dose studies are either carried out retrospectively, experimentally using anthropomorphic phantoms, or computationally. When performed experimentally, it is helpful to combine them with simulations validating the measurements. In this paper, we show how such a dose simulation method, carried out together with actual X-ray experiments, can be realized to obtain reliable organ dose values efficiently.

**Methods:**

A Monte Carlo simulation technique was developed combining down-sampling and super-resolution techniques for accelerated processing accompanying X-ray dose measurements. The target volume is down-sampled using the statistical mode first. The estimated dose distribution is then up-sampled using guided filtering and the high-resolution target volume as guidance image. Second, we present a comparison of dose estimates calculated with our Monte Carlo code experimentally obtained values for an anthropomorphic phantom using metal oxide semiconductor field effect transistor dosimeters.

**Results:**

We reconstructed high-resolution dose distributions from coarse ones (down-sampling factor 2 to 16) with error rates ranging from 1.62 % to 4.91 %. Using down-sampled target volumes further reduced the computation time by 30 % to 60 %. Comparison of measured results to simulated dose values demonstrated high agreement with an average percentage error of under $10 \%$ for all measurement points.

**Conclusions:**

Our results indicate that Monte Carlo methods can be accelerated hardware-independently and still yield reliable results. This facilitates empirical dose studies that make use of online Monte Carlo simulations to easily cross-validate dose estimates on-site.

## Introduction

Due to the growing number of fluoroscopically guided interventions (FGI), the importance for X-ray dose management has increased [1–3]. In the vast majority of cases, the anticipated medical benefit to the patient far outweighs any potentially high radiation exposure and associated risks. Nevertheless, assessing the applied dose critically is helpful to take preventive actions to avoid or treat radiation-induced injuries, if possible. While diagnostic reference levels [4] serve as a guidance for the expected overall exposure of the patient, it is difficult to draw conclusions on organ dose levels from them. However, for complicated FGIs, e.g., neuro-interventional procedures, repeatedly acquired digital subtraction angiography acquisitions, cone-beam CT reconstructions, and biplane imaging can lead to high dose values [5–9]. They may cause DNA damage [10] or cataract [11]. To alert physicians of potential deterministic injuries, such as local skin rashes or hair loss [6, 12–14], today’s interventional X-ray systems are equipped with skin entrance dose tracking techniques based on a patient model [15].

However, in contrast to skin dose monitoring, assessing organ dose requires much more prior information about the patient anatomy. Sophisticated algorithms are needed to solve the photon transport equation, such as Monte Carlo (MC) methods [16], finite differences [17], or deep-learning-based approaches [18]. While the task group report 195 (TG-195) of the American Association of Physicists in Medicine (AAPM) proposes a protocol to ensure the validity of such algorithms [19], uncertainties concerning patient anatomy and alignment, or material composition remain [20, 21]. Unfortunately, the high inter- and intra-procedure variance of organ dose values concerning complicated FGIs, therefore, demands for individualized patient-specific dose metrics [22, 23] and procedure-specific dose studies. Consequently, for most procedures, a high uncertainty remains when estimating organ or effective dose from the total air kerma using either simulation or experimental phantom studies [21, 24, 25].

If carried out correctly, both simulations and measurements can yield reliable reference values. However, they need to be conducted carefully to arrive at meaningful results. Experimentally obtained measurements are, for example, prone to discretization errors since there is usually only a limited number of dosimeters available and, because there is only limited time to do all measurements, a rather coarse sampling is unavoidable. In addition to the systematic errors caused by the coarse sampling, it is also challenging to reproducibly carry out sufficiently many measurement series to minimize stochastic uncertainties. In contrast, simulation approaches are easy to repeat, but it is difficult to model the imaging setting accurately. This is why we propose to combine both approaches to arrive at a more accurate result by leveraging the specific advantages of each technique [21]. However, joint experimental and computational studies often require manual effort to adapt simulation parameters and to properly account for the imaging settings. In other words, it can be very tedious to conduct them, in particular, when MC simulations take so long that they are only available afterward.

To facilitate such combined studies, we propose a framework with which to carry out associated MC simulations during experimental measurements on-site: XDose. The idea behind XDose is to avoid any manual parameter fine-tuning by utilizing a Jacobian inverse kinematics solver yielding position, and orientation of each tracked object in the test suite entering the MC simulation. The X-ray system itself provides physics parameters such as the air kerma and the tube voltage as inputs to the MC simulation. To keep the computational complexity of the MC code manageable, we apply a recently presented filtering-based variance reduction technique [26].

## Materials and methods

**Fig. 1:**
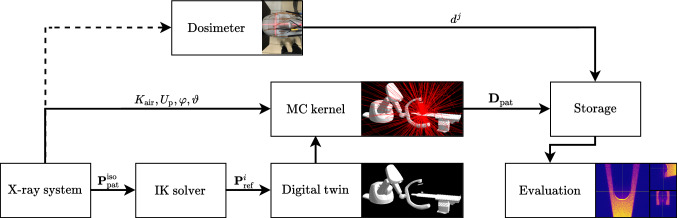
Overview of the XDose workflow. The X-ray system provides the spatial relationship between the X-ray source and the assumed phantom position ${{\varvec{P}}}_\text {pat}^\text {iso}$ in the patient/table coordinate system. Using an inverse kinematics (IK) solver, we can find the position and the orientation of each system component and the phantom ${{\varvec{P}}}_\text {ref}^i \in \text {SE}(3)$ in a shared reference coordinate system. Based on the known imaging geometry, air kerma $K_\text {air}$, tube peak voltage $U_\text {p}$, and X-ray opening angles $\varphi$ and $\vartheta$, a MC simulation is carried out that yields a noisy patient dose estimate. Our model-based filtering eventually gives a smooth dose distribution inside the patient ${{\varvec{D}}}_\text {pat}$. In addition to the computational dose estimation, we simultaneously measure the applied dose using MOSFET dosimeters at predefined measurement points $d^j$

Figure 1 depicts an overview of the XDose framework. Based on the internal messaging protocol of an X-ray system, the spatial relationship between the X-ray tube, flat-panel detector, and the assumed patient model position is read out. Using an in-house inverse kinematics solver, a digital twin of the imaging setting, including a patient model, is created. The MC kernel uses this digital twin and additional parameters provided by the X-ray system to estimate a coarse dose distribution inside of the patient model. Our filtering approach is then used to reconstruct a smooth dose distribution, which can be compared locally to dose values experimentally measured using MOSFET X-ray dosimeters. In the following, we describe the computational dose estimation.

### Computational dose estimation

#### Model of the imaging setting

An accurate geometric model of the imaging setting is at the heart of our computational dose estimation pipeline. The X-ray system continuously streams the current isocenter coordinate system ${{\varvec{P}}}_\text {pat}^\text {iso} \in \text {SE}(3)$ in patient coordinates. Based on ${{\varvec{P}}}_\text {pat}^\text {iso}$, our in-house inverse kinematics solver code calculates the position and orientation of each system component ${{\varvec{P}}}_\text {ref}^i \in \text {SE}(3)$ in the reference or world coordinate system. Given a patient model placed on the system’s patient table and registered, its position and orientation get updated implicitly. Multiple sources for such a patient model are conceivable: (a) shape and organ meshes based on meta-parameters [27], (b) segmented and labeled CT scans [28, 29], or (c) established reference phantoms as proposed by the ICRP [30] or the XCAT family [31]. Since we are currently using this prototype for phantom studies only, we manually aligned the phantom on the patient table based on distinct features. For future applications in a clinical setting, a robust registration step is needed.

#### Monte Carlo simulation

Our MC code is based on the Geant4 toolkit [32]. To provide physical plausibility, we employed the Geant4 configuration used in the TG-195 report [19] and a previous study [21]. Besides accurately capturing the spatial relationship between all components of the imaging settings, mapping the physics’ characteristics is critical to obtain accurate results in the MC simulation. Assuming a tungsten anode with $2.3\,\hbox {mm}$ aluminum-equivalent inherent filtration, we calculated the X-ray spectrum based on the tube peak voltage $U_\text {p}$ and copper pre-filtration using in-house software (Siemens Healthcare GmbH, Forchheim, Germany) based on Boone’s algorithm [33]. To account for X-ray beam collimation, the associated cone opening angles $\varphi$ and $\vartheta$ were determined based on system settings. Primary dose and scatter dose were scored separately inside the patient model and at the interventional reference point. The ratio between the air kerma $K_\text {air}$ provided by the X-ray system and the estimated primary dose at the interventional reference point was used to scale the calculated dose distributions.

#### Filtering-based variance reduction

Unfortunately, the flexibility of Geant4 requires to run it on a CPU. Although there exist GPU-accelerated photon and electron transport codes [16], they provide rather rigid interfaces to define experimental setups. To still arrive at smooth results in a reasonable run-time, filtering-based variance reduction techniques can be used. Perona–Malik anisotropic diffusion [34, 35] and Savitzky–Golay filtering [36, 37] have both been applied successfully to the denoising of coarse MC simulations. Also, down-sampling of the target volume is a common approach to reduce the simulation time. To cross-validate computed dose values and measured results obtained at discrete spatial positions, a high-resolution dose distribution is needed. Based on the concepts of down-sampling and filtering, we recently presented a similar strategy to speed up MC simulations [26]. However, instead of directly applying a denoising algorithm to coarse MC simulations, we first proposed to down-sample the target volume to further reduce the number of primary particles needed to arrive at an acceptable accuracy [26]. The down-sampling was performed by grouping neighborhoods of voxels to hybrid mixture materials based on the fraction of mass (FoM) of each individual voxel. In a proof-of-concept study, we showed that this down-sampling could be inverted for dose distributions using the 2-D guided filter [38] and a voxelized absorption guidance map based on the patient model. Following the FoM, down-sampling of a neighborhood $\mathscr {N}$ in the target volume ${{\varvec{f}}}$ to a single voxel ${{\varvec{f}}}^\prime _\mathscr {N}$ is given by

1 $$\begin{aligned} {f}^\prime _\mathscr {N}= & {} \frac{\sum _{x\in \mathscr {N}} V \cdot {\rho }_x \cdot {f}_x }{\sum _{x\in \mathscr {N}} V \cdot {\rho }_x} = \frac{\sum _{x\in \mathscr {N}}{m}_x {f}_x}{\sum _{x\in \mathscr {N}}{m}_x} = \sum _{x\in \mathscr {N}} \frac{{m}_x}{m_{\mathscr {N}}} {f}_x\nonumber \\= & {} \sum _{x\in \mathscr {N}} w^\text {FoM}_x {f}_x , \end{aligned}$$

with the voxel volume *V* [$\hbox {cm}^{3}$], mass density $\varvec{\rho }$ [$\hbox {g}\hbox {cm}^{-3}$], mass ${{\varvec{m}}}$ [$\hbox {g}$], the FoM weight $w^\text {FoM}$, and the linearized voxel index *x*. Unfortunately, the straightforward implementation of this idea in Geant4 scales exponentially with the resolution of the target volume rendering the approach impracticable [26]. Therefore, this method cannot be applied to real-world scenarios. However, the FoM down-sampling merely relates to the weighted average or expected value of the neighborhood $\mathbb {E}_w[{{\varvec{f}}}]$. Since, for the human anatomy, we encounter mostly uniformly or logarithmic-normally distributed neighborhoods of voxels, we can approximate the (weighted) average value with the associated statistical mode $\mathbb {M}$. Using the mode, we can ensure that at least the most often occurring tissue is well represented.

**Fig. 2:**
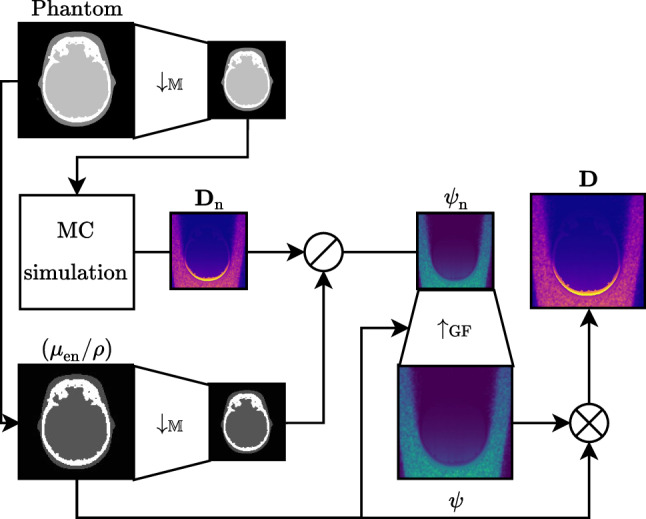
Filtering algorithm used to reconstruct smooth dose distributions ${{\varvec{D}}}$ from coarse ones ${{\varvec{D}}}_\text {n}$ (see Fig. 1). First, the coarse dose distribution ${{\varvec{D}}}_\text {n}$ is simulated based on a down-sampled patient model (Phantom). Element-wise division $\oslash {}$ of ${{\varvec{D}}}_\text {n}$ by the down-sampled mass-energy absorption $(\varvec{\mu }_\text {en}/\varvec{\rho })_\text {n}$ yields a coarse estimate of the photon energy fluence $\varvec{\psi }_\text {n}$. Using a guided filter with $(\varvec{\mu }_\text {en}/\varvec{\rho })$ guidance, we obtain the smooth high-resolution photon fluence $\varvec{\psi }$. Element-wise multiplication $\otimes {}$ with $(\varvec{\mu }_\text {en}/\varvec{\rho })$ yields the associated dose distribution ${{\varvec{D}}}$

Figure 2 depicts an overview of the resulting simulation and 2-D slice-wise filtering approach. Based on the assumption that the macroscopic properties of X-rays are approximately equal in small neighborhoods, we down-sample the target volume using the statistical mode $\downarrow _\mathbb {M}$. In the isotropic 2-D case, the neighborhood $\mathscr {N}$ is a quadratic window of size *s*. MC simulation of this volume yields the coarse dose distribution ${{\varvec{D}}}_\text {n}$. To reconstruct a high-resolution dose distribution ${{\varvec{D}}}$ from this coarse ${{\varvec{D}}}_\text {n}$, we apply our proposed up-sampling scheme [26]. Although the human anatomy comprises both homogeneous as well as anatomically diverse areas, in dosimetry applications averaged interaction coefficients and mass densities are typically used. This is why we further assume a charged particle equilibrium (CPE), under which the absorbed dose ${{\varvec{D}}}$ [$\hbox {J}\hbox {g}^{-1}$] in a volume corresponds to the collision kerma ${{\varvec{K}}}_\text {col}$ [$\hbox {J}\hbox {g}^{-1}$] for low-energy X-rays and relates proportionally to the photon energy fluence ${\varvec{\psi }}$ [$\hbox {J}\hbox {cm}^{-2}$]:2with the mass-energy absorption coefficient $(\varvec{\mu }_\text {en} / \varvec{\rho })$ [$\hbox {cm}^{2}\hbox {g}^{-1}$]. This relationship allows us to decouple material properties and absorbed dose. Thus, we transform the coarse dose distribution ${{\varvec{D}}}_\text {n}$ to its associated photon energy fluence $\varvec{\psi }_\text {n}$, before applying guided filter up-sampling $\uparrow _\text {GF}$ [26, 38]. The overall denoising step is defined by

3 $$\begin{aligned} {{\varvec{D}}} = \left( \frac{\varvec{\mu }_\text {en}}{\varvec{\rho }}\right) \cdot \uparrow _\text {GF}\left( \left( \frac{\varvec{\mu }_\text {en}}{\varvec{\rho }}\right) , \frac{{{\varvec{D}}}_\text {n}}{\downarrow _\mathbb {M}\left( \varvec{\mu }_\text {en}/\varvec{\rho }\right) }, r \right) , \end{aligned}$$

with the filtering radius *r*. Although variable, in the following, the radius is dependent on the down-sample window size *s* and defined as $r(s) = \lfloor 0.5 s \rceil + 1$, where $\lfloor \cdot \rceil$ denotes rounding to the next integer value.

#### Experiments

To be comparable to our proof-of-concept study, we used the same extent from the Visible Human (Vishum) [39] used before [26]. Originally, the Vishum phantom has an axial resolution of $512 \times 512$ voxels with $0.91\,\hbox {mm} \times 0.94\,\hbox {mm} \times 5\,\hbox {mm}$ spacing. Each phantom organ label is either assigned to air, soft tissue, adipose tissue, or bone tissue. Down-sampling was performed slice-wise by $s \in \{2, 4, 8, 16\}$ using the statistical mode. In a first experiment, to assess the general reconstruction capabilities of our method, we simulated dose distributions comprising $10 \times 10^{8}$ primary photons sampled from a 120 kV peak voltage spectrum for all down-sampled phantoms. As a reference, we performed a simulation of the original spatial scale with $20 \times 10^{8}$ primary photons and otherwise identical parameters. The voxel-wise statistical uncertainty ($2\sigma$) was in the range of 4.9 % to 19.6 % (X-ray entrance to exit) for $10 \times 10^{8}$ primary photons, and in the range of 3.4 % to 13.7 % for $20 \times 10^{8}$ primary photons, respectively.

### Experimental dose estimation

#### Anthropomorphic phantom

We used the anthropomorphic ATOM phantom (ATOM Adult Male Model 701, Computerized Imaging Reference Systems, Inc., Norfolk, VA, USA) to emulate in vivo dose measurements. It consists of 39 slices with $2 \hbox {cm}$ thickness of tissue-equivalent materials for average bones, lung, brain, and soft tissue (deviation with respect to the linear attenuation is 1 % to 3 % in the energy range of $50 \hbox {keV}$ to $15\,\hbox {MeV}$). To create the same conditions in the simulation, we scanned the phantom using a CT system (SOMATOM Definition Edge, Siemens Healthcare GmbH, Forchheim, Germany) and reconstructed it with a $0.6 \times 1.0 \times 1.0\,\hbox {mm}$ voxel size. Therefore, one physical slice corresponds to approximately 33 digital slices. Afterward, we segmented and labeled the reconstructed CT volume using thresholding and manual corrections.

**Fig. 3:**
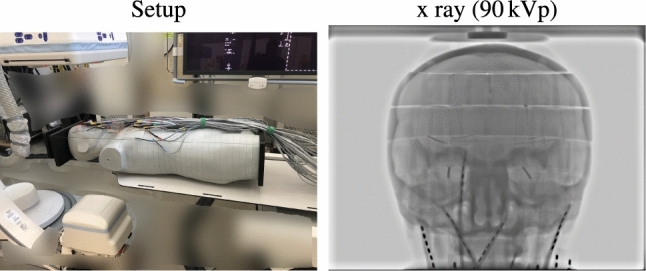
Experimental setup and one corresponding X-ray projection image. To avoid uncertainties due to the table material, we place the phantom in a way that the table was not in the field of view when taking dose measurements. We investigated positioner primary angles of $0 ^{\circ }$ (posteroanterior), $45 ^{\circ }$, and $90^{\circ }$ (lateral), which are commonly used in neuro-interventional procedures

**Fig. 4:**
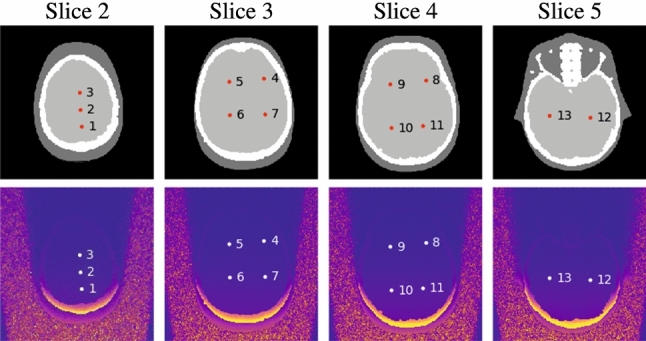
Measurement points inside the brain taken for the experimental as well as for the computational study and associated examples of dose distribution overlays estimated using MC simulation

#### MOSFET dosimeters

For experimental dose measurements, we used 13 high-sensitivity metal oxide semiconductor field effect transistor (MOSFET) dosimeters (TN 1002RD-H, Best Medical Canada Ltd., Ottawa, ON, Canada). To enable online monitoring, up to five probes can be hooked up to a mobile readout system (mobileMOSFET system, model TN-RD-70-W, Best Medical Canada Ltd., Ottawa, ON, Canada). The mobile readout system can be connected to a computer via a Bluetooth wireless transceiver and an associated remote monitoring dose verification software. The same software is used to calibrate the MOSFET probes individually. To this end, each probe is irradiated with a pre-defined reference dose level. The reference dose level is estimated using a $530\,\hbox {cm}^{3}$ ionization chamber (PM500-CII 52.8210, Capintec Inc., Ramsey, NJ, USA) in combination with the Unidos dosimeter (PTW, Freiburg, Germany). The ionization chamber is biannually calibrated by PTW accredited by the German National Accreditation Body (D-K 15059-01-00) as a calibration laboratory in the German calibration service (Deutscher Kalibrierdienst). Based on the reference dose value, the monitoring software estimates a calibration factor for each MOSFET probe. The manufacturer ensures an uncertainty under 0.8 % to 3 % in the range of 20 cGy to 200 cGy. The manufacturer also specifies an angle-dependent uncertainty of $2 \%$, which, however, is negligible concerning the overall uncertainty for low exposure of the probes.

#### Experiments

To assess our variance reduction method, we first analyze how well our general simulation framework is applicable to experimental measurements using the ATOM phantom. To this end, we set up an experiment that is fully reproducible in our XDose MC framework. Since the ATOM phantom only allows for rather coarse and discrete dose sampling patterns, we decided to focus on the brain, an important large and homogeneous organ. Therefore, we centered our experiments around neuro-interventional applications. This also allowed us to remove the table from the field of view/irradiation (see Fig. 3).

**Fig. 5:**
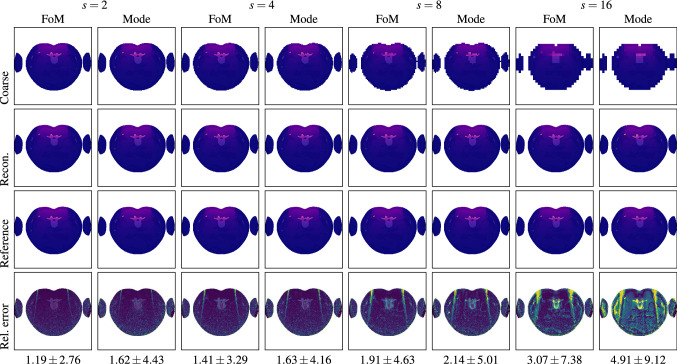
Low-resolution dose distributions using either an artificial down-sampling [26] based on the fraction of mass (FoM) or the implemented down-sampling based on the statistical mode (Mode), their associated reconstructed high-resolution counterparts (Recon.), and the percentage error maps (Rel. error) with respect to the ground truth dose distributions (Reference). The dose distributions are scaled to 0 mGy to 500 mGy (dark blue over purple to yellow); the error maps are in the range of 0 % to 30 % (dark blue over green to yellow). The associated average absolute percentage errors are also given for each error map. The parameter *s* denotes the neighborhood size used for down-sampling. For example, $s = 2$, means $2\times 2$ neighborhood

From a clinical point of view, our example is also relevant as, brain tissue is at certain risk for late tissue reactions and (deterministic) effects [40]. Although not common, in complex interventional procedures brain radiation doses above the ICRP absorbed dose threshold of $500\,\hbox {m}\hbox {Gy}$ have been reported [41, 42]. In addition, focusing on a large and mostly homogeneous organ gives an interesting comparison between measuring discrete dose values and simulating continuous dose distributions. We used the same X-ray system (Artis zeego, Siemens Healthcare GmbH, Forchheim, Germany) for all measurements. In total, we covered three standard neuroradiology projection angles, $0 ^{\circ }$, $45 ^{\circ }$, and $90 ^{\circ }$, relating to posteroanterior, oblique, and lateral view directions [43]. Furthermore, two peak tube voltages ($70\,\hbox {k}\hbox {V}$p, $1.31\,\hbox {mm}$ Al air kerma half-value layer, and $90\,\hbox {k}\hbox {V}$p, $1.68 \hbox {mm}$ Al air kerma half-value layer) were used in our experiments. The C-arm’s isocenter was aligned with the center of the phantom’s head, the source-to-isocenter distance was $80 \hbox {cm}$, and the source-to-image distance was $120\,\hbox {cm}$, respectively. For each imaging setting, we irradiated the phantom with $100\,\hbox {m}\hbox {A}$ tube current for $20\,\hbox {s}$ with 30 frames per second to ensure sufficient exposure of all MOSFET probes; neither pre-filtration nor collimation was applied. To account for the MOSFET uncertainty and to ensure stable average dose values for each measurement point, we repeated each acquisition five times. After each irradiation, we waited $5\,\hbox {min}$ to ensure total discharge of the MOSFET probes. Therefore, the measurement protocol took $26\,\hbox {min}$ and $40\,\hbox {s}$ (including $5\times 20\hbox { s}$ acquisition time) for one imaging setting, leaving sufficient time to run and finish our simulation in parallel (online). The average air kerma was $49.28\pm 0.07 \hbox {m}\hbox {Gy}$ for the $70\,\hbox {k}\hbox {V}$p spectrum and $84.66\pm 0.10\,\hbox {m}\hbox {Gy}$ for the $90\,\hbox {k}\hbox {V}$p spectrum, respectively. The MOSFET probes were placed inside the ATOM phantom as shown in Fig. 4. To affix the MOSFET probes, we encased them in soft tissue-equivalent holders of the same size and shape of the drillings in the phantom. Since this experiment focused on the overall agreement of the computational and the experimental approach, we carried out the associated simulations offline using $25 \times 10^{8}$ primary photons and the high-resolution digitized phantom.

## Results

We evaluated our approach from different perspectives. First, we compared our results using the statistical mode as a down-sampling operator to our previous proof-of-concept study [26]. Second, we assessed the potential to accelerate MC simulations using our filtering-based variance reduction technique. We also point out sensible threshold values to obtain meaningful dose estimates online, meaning in the same time frame needed to collect all measurements, e.g., 25 minutes. Third, we compared computationally estimated to experimentally measured dose values using MOSFET dosimeters and the anthropomorphic ATOM phantom to find how well both methods agree.

**Fig. 6:**
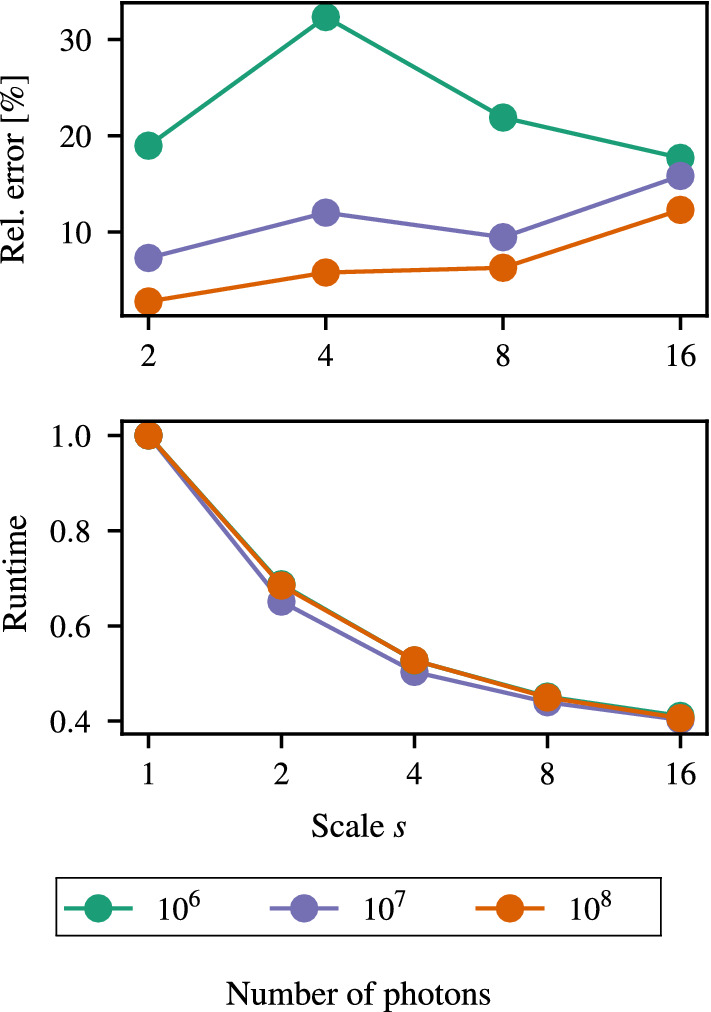
Performance measures of our approach for different scales *s* and number of primary photons simulated. Top: Relative error compared to the low-uncertainty reference dose distribution. Bottom: Normalized runtime as function of the down-sampling scale *s*. All data refer to our method using the Mode down-sampling strategy. Note that the data for $10^{6}$ photons is superimposed by the data for $10^{8}$ photons in the bottom figure

**Fig. 7:**
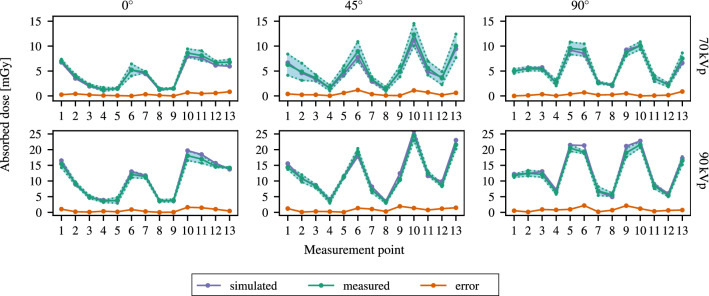
Measured dose values, simulated dose values, and the associated absolute errors per measurement point for each imaging setting. The simulations were scaled to the air kerma measured at the interventional reference point. The area enclosed by dotted lines indicates the $2\sigma$ confidence interval of the measurements

### Computational dose estimation

Figure 5 shows the initial results of the proof-of-concept study (FoM) [26] and the results of our refined method (Mode) accompanied by the associated average error rates. Note that, previously, we considered only $10\times 10^8$ primary photons as ground truth, which yielded slightly higher error rates [26]. Also, the low-resolution dose distributions for the FoM method were obtained by directly down-sampling high-resolution dose distributions, while we actually used down-sampled target volumes for the Mode approach. The FoM approach slightly outperformed our actual implementation, however, only by a small margin. For the FoM method, we observed a scale-dependent error of 1.19 % to 3.07 %, while for our implementation, the error ranged from 1.62 % to 4.91 %. For spatial scales *s* of 2 to 8, high errors above $10 \%$ occurred mostly outside of the primary X-ray, while for $s=16$, the overall error distributions indicate a systematic trend. Figure 6 shows plots of the relative error and runtime performance measures of our method for a reduced number of simulated primary particles ranging from $1 \times 10^6$ to $1 \times 10^8$ for different scales *s*. Note that for $1 \times 10^7$ and $1 \times 10^8$ particles, the error increased with increasing the spatial scale *s*, while for fewer particles, the error decreased again with $s = 8$ and higher.

### Acceleration potential

To assess the potential to accelerate MC simulations using our method, we compared the execution time for different down-sampling scales $s \in \{2, 4, 8, 16\}$ and number of particles $N \in \{10^6, 10^7, 10^8\}$. In general, we observed an exponential acceleration by up to $60 \%$ with doubling the spatial scale *s* for an arbitrary number of simulated primary photons (see Fig. 6). The average baseline runtimes ($s=1$) were $236\,\hbox {s}$, $2328\,\hbox {s}$, and $23574\,\hbox {s}$ for $1 \times 10^6$ to $1 \times 10^8$ particles, respectively. All simulation runs were carried out using a single Intel Xeon E3-1240 processors with four physical cores and hyperthreading for parallelization. The execution time of the down-sampling and up-sampling scheme is negligible. The measured runtimes include the initialization of Geant4, which took approximately $6\,\hbox {s}$. Depending on the desired uncertainty of the MC simulation, our method, therefore, enables online computational dose estimation. For instance, a simulation of $1 \times 10^{7}$ primary particles can be carried in under $10\,\hbox {min}$ using down-sampling by $s=8$ and only yields $10 \%$ uncertainty.

### Experimental dose estimation

Figure 7 summarizes the experimental dose values and the simulated dose values at the measurement points specified in Fig. 4. The simulated dose values were scaled according to the associated average air kerma of the measurements. Overall, we achieved a high agreement between experimentally and computationally estimated dose values at the measurement points for both spectra and all three projection angles. The estimated dose values using MC simulation were inside the confidence interval ($2\sigma$) of the MOSFET measurements for most measurement points and experimental setups (see Fig. 7).

**Fig. 8:**
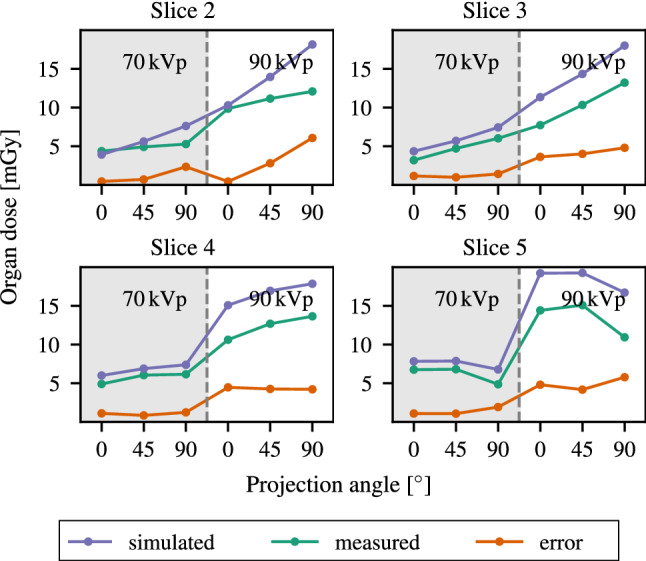
Brain-equivalent dose values for selected slices of the phantom (see Fig. 4) calculated based on either a MC simulation or obtained using empiric measurements, respectively

Figure 8 shows plots of the slice-wise brain-equivalent dose averaged over organ volume ($w_\text {R} = 1$) for each experiment setup. These values were obtained either from discrete measurements dose values or simulated, continuous dose distributions. While the overall trend in both approaches was similar, the measured organ-equivalent dose values underestimated the simulated ones by up to 20 % to 50 %. This finding substantiates our assumption that combining empiric measurements with computational methods is necessary to obtain accurate and representative organ dose values.

## Discussion

Radiation exposure during X-ray examinations should always be as low as reasonably possible to prevent any risk to either the patient or any other involved personnel. Metrics for specific diagnostic and interventional procedures can be retrospectively determined based on experimental measurements or computational studies. Due to the high inter- and intra-procedure variability for complicated FGIs, it is, however, difficult to establish a general protocol to acquire reference organ dose values. For example, it can be difficult to accurately map clinical imaging settings to MC simulations unless the underlying X-ray physics as well as the patient’s anatomy and position relative to the X-ray device are known. Empiric methods often lack reproducibility and are prone to measurement uncertainties [21].

To arrive at consistent results, we, therefore, developed a framework to combine both approaches using a self-validating workflow—XDose. XDose integrates an in-house inverse kinematics solver, the simulation toolkit Geant4, and an optimized filtering-based variance reduction technique.

Concerning the computational dose estimation, we found that our refined variance reduction technique was on par with the one from a previous study [26]. We were able to reconstruct high-resolution dose distributions from coarse ones with an average absolute percentage error in the range of 1.62 % to 4.91 % for down-sampling factors $s \in \{ 2, 4, 8, 16\}$. Error rates above $10 \%$ mostly occurred outside of the primary X-ray and at interfaces of different tissues, which, however, are negligible for organ equivalent dosimetry. Only for heavy down-sampling of the target volume ($s=16$), we observed such errors over the whole target volume. With decreasing the number of primary photons to $1 \times 10^8$ to $1 \times 10^6$, the percentage error increases to 2 % to 20 %, depending on the spatial scale *s*. With increasing down-sampling factor, the computation time of the MC simulation dropped by 30 % to 60 % regardless of the number of primary particles. For instance, we found that a simulation of $1 \times 10^{7}$ primary particles could be carried out in under $10 \hbox {min}$ with only $10 \%$ uncertainty using our down-sampling and super-resolution approach. Our method can thus be used to simultaneously measure and compute dose values with low uncertainty, as our measurement protocol takes approximately $25\,\hbox {min}$ for one imaging setting.

To compare our computational framework to our experimental setup, we carried out both measurements and simulations for six different imaging settings of the head of an anthropomorphic phantom tailored for patient dosimetry. Overall, we found a strong correlation between physical and computational measurement points for all settings. This shows that, XDose can be used to facilitate the estimation of organ-equivalent dose values. These values can be simultaneously cross-validated or calibrated using empirical measurements, without the need for potentially error-prone manual parameter configurations. As such, XDose has the potential to complement anthropomorphic phantom studies with accurate MC simulations.

Since our variance reduction method relies on down-sampling of the target volume, it is best suited for estimating the dose in rather compact or convex organs. To what extent XDose can be used for dosimetry related to interfaces, e.g., skin, or small organs, boils down to a trade-off between simulation accuracy and acceleration. Our current approach is based on classifying voxels into four major tissue types to reach a good trade-off between computational complexity and performance. This is a general simplification often made in dosimetry. Future studies are needed to investigate its application to more diverse tissue models.

Another important piece of future work is an in-depth comparison between measured values and our method. Since, for organ-equivalent dose estimation, we can safely assume mostly homogeneous regions of interest, merely tracking of primary photons ignoring secondary electrons might be accurate enough for X-rays in the diagnostic energy regime. Also, the integration with traditional variance reduction techniques such as Woodcock tracking [44], or super Woodcock voxels [45] is conceivable. With additional models, the scope of XDose can be easily extended to the whole interventional suite, including peripheral devices and consequently staff dose estimations.

## Conclusion

We proposed a filtering-based variance reduction approach to speed up Monte Carlo simulations for interventional procedures to facilitate the on-site combination of computational and experimental methods. The performance of our down-sampling and filtering-based variance reduction technique demonstrated that empiric measurements and associated simulations can be performed simultaneously in the same setting. This combination has the potential to facilitate a smooth workflow for estimating organ dose values or cross-validation of measurements and simulations.
